# New Applications of Vulcanized Rubber

**Published:** 1862-04

**Authors:** J. Richardson


### NEW APPLICATIONS OF VULCANIZED RUBBER.

BY DR. J. RICHARDSON.

THE facility with which unvulcanized rubber may be molded into almost any conceivable form, and the indestructible nature of this substance when indurated, would seem to suggest at once its peculiar adaptation to the various necessities of the dentist. Within the past few years, it has come into very general use as a base for artificial teeth, and such has been its apparent fitness for the purpose, that perhaps four-fifths of the profession in this country have been induced to use it. It has been recommended and used in the construction of mechanical appliances for the correction of irregularities in the arrangement of the natural teeth, for which purpose it undoubtedly possesses many advantages. More recently, it has been employed to unite porcelain teeth to a metallic or swaged base. I regard the latter as one of the most important and useful applications of vulcanized rubber which has yet been suggested, and one which, when it is better and more generally understood, will in a great measure supersede the usual method of attaching the teeth to the plate by means of solder. Recently, I have extended its application to the following purposes, for which it seems well adapted:

HANDLES FOR PLUGGING INSTRUMENTS.—The only application I have made of the rubber for this purpose has been to small pointed pluggers, formed from wire-handled excavators. The entire shaft of the instrument, with the exception of the polished portion next the point, is closely wrapped with a layer of rubber cut just wide enough to encircle it, adding a second and third in the same manner, or as many as may be necessary to give it the required thickness. Two layers, when vulcanized and dressed down with files to the octagonal form (which I prefer), will be large enough for small points. Any number of instruments being encased in this manner are imbeded in plaster and vulcanized. The temperature employed for this purpose I think does not materially draw the temper—at any rate, the heat necessary to re-temper need not affect the rubber which is at some distance from the point.

Handles formed from this substance have a much more pleasant feel in the hand of the operator than uncovered steel, or even than ebony or ivory, and are not liable to the same objections which may be preferred to leather, since they do not absorb either the secretions of the mouth or moisture from the hands, and are, therefore, when polished, easily kept clean, not only in appearance but in fact.

Pivoting Teeth.—I propose, in all practicable cases, to substitute vulcanized rubber for wood in pivoting teeth as ordinarily practiced. Take small gold wire (alloyed with platinum), say about the dimensions of a medium sized knitting needle, or about one-third the diameter of the enlarged opening in the root, encase closely in a single layer of rubber, and vulcanize, but not so hard but that it shall possess some degree of flexibility, to admit of any necessary slight change in the direction of the pivot when permanently adjusting the crown. The relative advantages of the rubber pivot traversed with wire I think are obvious. It is wholly impervious to the fluids of the mouth,—it will present greater resistance to the forces applied in mastication than either the crown or root, and will sustain the pivot-crown immovably in its place. Wood readily absorbs and becomes thoroughly saturated with the secretions, and it is chiefly from this source that the offensive odor so frequently associated with pivot teeth arises. Besides, wood being flexible, its fibres are soon broken up or detached by the mobility of the crown, whose base is seldom accurately fitted to the root, thus necessitating a frequent renewal of the pivot.

*Wired Rubber Pivot, with Rubber interposed between the Crown and Root.*—The object of this method is to secure a faultless adaptation of the base of the crown to the filed extremity of the root, and by interposing one or two thicknesses of gold foil, to render the joint impervious to the fluids of the mouth. Having prepared the root in the ordinary manner, select and fit the pivot-crown to the vacuity, leaving something of a space posteriorly between the base of the crown and the root. Fit a wood pivot to the enlarged opening in the root, accurately, but in such manner that it may be easily withdrawn—the end projecting from the root should be trimmed down to say half the diameter of the hole in the crown, so that when the latter is applied in the manner to be mentioned directly, some margin will be left, admitting of a proper adjustment of the crown when applied to the space. With the wood pivot in the root, fill the hole in the crown with stiffened wax, warm the pivot crown sufficiently to soften the wax, and apply it to the root in the desired position with respect to the other teeth. When the wax has hardened somewhat, withdraw the crown carefully on a line with the pivot, bringing the latter with it; then add sufficient softened wax to the base of the crown to fill in the gap or space between the crown and root; replace the tooth with the pivot attached, and press up until the crown again takes its proper position in the vacuity. By this means we get an *impression* of the filed extremity of the root, and at the same time secure an accurate relation of all the parts. The crown and pivot are then carefully removed. Now, take plaster and pour a small quantity on a piece of paper or card and sink the pivot into it until the surface of the wax at the base of the crown rests upon the plaster, and then build the latter up upon the front part of the crown to the cutting edge, thus forming a shallow bed for its anterior face. When the plaster has hardened, warm the model to soften the wax, and then remove the crown and wax,—the pivot will be found remaining in the model, but which, if previously oiled, can be readily drawn. We have now in the model an accurate representation of the end of the root, the size and direction of the fang canal, and, when the crown is replaced in its shallow bed, also the space between the crown and root to be filled in with rubber. The hole in the model, corresponding with that in the root, should be enlarged somewhat, and this may be done with the same drill used in enlarging the orifice of the fang. After varnishing the hole in the model, pack in softened rubber until full, and insert into this the gold wire previously heated, one end projecting a line or so. Next fill the hole in the crown with rubber, heat the crown, and press it down upon the model and over the gold wire until the crown goes accurately into the depression made for it in the model. Then pack softened rubber into the space between the base of the crown and the model until it is filled in compactly. The whole is then encased in plaster, confined in a flask and vulcanized. If the foregoing manipulations are carefully conducted, the crown will go to its place in the mouth with unerring accuracy, and by placing one or two thicknesses of gold foil between the rubber and root, the joint will be rendered impervious. There is still an additional advantage in this method. All are aware of the liability of a wood pivot drawing from the crown where the latter is short and the hole in it shallow. The rubber pivot, vulcanized in the crown, I do not believe can be drawn from the shallowest cavity without fracturing the crown. I submitted this test to members of the Cincinnati Association, none of whom, holding the crown in the fingers, could withdraw the pivot with pliers, the hole being a fraction over a line in depth. I subsequently fixed the same tooth in a vise, and in drawing the pivot with pliers, fractured the crown in the attempt. In very many cases, this circumstance is of great value.

*Canal of Fang lined with Rubber Tube for the reception of*

*Gold Pivot Vulcanized into a Crown with a Rubber Base.*—The manner of forming a rubber tube for the fang is exceedingly simple. Draw a wire of gold alloyed with platinum, say half the ordinary diameter of enlarged opening in the root, and from two to three inches in length; encase this in a single lamina of thin tin leaf, to prevent the rubber from adhering to the wire, and which will enable the latter to be readily withdrawn after induration of the gum. Then wrap the wire tightly in a single layer of rubber, encase in plaster and vulcanize; place the vulcanized tube in a vise, and withdraw the wire with pliers. Digest the tube in solution of muriatic acid to remove tin. The same wire is used for pivot, and will enter the tube readily but accurately. Dress down a portion of the tube to fit the orifice in the fang, and force the tube up with wire encased, and then remove the latter, and file off the tube to the end of the root. The tube may be fixed more securely in the fang by smearing its surface with a little thin osteoplastic material before introducing. The crown being fitted to the space, and the gold pivot placed in the tube with a line or more projecting, the hole in the crown is filled with wax, and applied to the base of the crown, and the same manipulations practiced to secure an impression of the filed extremity of the root, as previously described. Remove the crown, with wire pivot attached, carefully from the mouth, and imbed in plaster precisely in the manner as before related. In this case, however, after the crown is removed from the model, the wire pivot is allowed to remain attached to the model, when the hole in the crown is filled with rubber, the latter softened with heat, and the crown forced back into its place upon the model, the projecting end of the wire pivot penetrating the rubber in the crown. The interspace is then packed as before, the whole encased and vulcanized. In reapplying the tooth to the fang in the mouth, the pivot will be found to enter the tube too freely to give it the proper support,—the latter may be secured, either by flexing the pivot upon itself slightly, or by splitting it part way with a fine watchspring saw, and spreading the ends apart, as recommended by Dr. Dwinelle. By this method, the patient will be enabled to remove the crown at will, for the purpose of cleansing the parts, though if the crown can be secured in such manner that there shall be no movement of it, or change of relation with the root, under any force that may be applied to it, (a practicable thing with this method) and the joint can be rendered perfectly impervious, I generally prefer to make the crown stationary, by fixing it immovably in the first instance.

*Substitution of a Plate for a Pivot Tooth.*—Line the canal with rubber tube as before. Adjust a plate tooth to the space, and then solder on a gold backing. Place the wire pivot in the tube, with the end projecting. Hold the crown firmly to its position in the vacuity, and press softened wax or plaster around the end of the pivot and down upon the filed surface of the root and against the gold backing. Remove crown and pivot carefully, and imbed in plaster and sand ; remove the wax or plaster, heat up and solder the pivot to the backing. Reapply to the mouth, press in softened wax posteriorly down upon the filed extremity of the root ; remove carefully ; imbed as before in plaster ; remove wax, and pack softened rubber down upon the portion of the model representing the filed end of the root, around the pivot and against the gold backing, adding sufficient gum to enable you to dress it, when vulcanized, to the form of the adjoining natural organs. I have recently so pivoted a plate tooth in a case which required it, and I regard it in every particular as the most skillful and serviceable operation of the kind I have ever performed.

Impression Cups.—Take a plaster model obtained from an impression of the mouth, and fill out where necessary with fresh plaster, and trim to the desired form. The same may be obtained from a block of plaster, but it is more convenient to use a model, many of which, of various forms and sizes, collect in the shop, and from which suitable selections may be made. To the model fit accurately one or two thicknesses of impression rubber sheeting, and attach a strip of the proper width and length to its lower and anterior portion, to represent the handle. Portions of wax may be added wherever necessary, to give it as nearly as possible the form of an ordinary impression cup. The model, with the rubber covering attached, is then placed in the lower section of the flask, and treated in the same manner as when constructing an artificial denture in a base of rubber. The sections of the flask separated and the rubber removed, the matrix is packed with the vulcanite material, and vulcanized in the usual way. The cup is then finished on the inside with a scraper, and its exterior surface neatly dressed and polished. If it is desired to duplicate the same form of cup, the one so finished may be used to procure the matrix in plaster, it being readily and quickly accomplished. It will be readily perceived that almost any conceivable form of cup for ordinary or extraordinary cases may be easily made by any one who has facilities for vulcanizing, and thus the operator has the means of providing himself with a set which will fulfill his various wants in this respect more perfectly than the metallic cups in general use, and which vary more in size than in configuration. They make a very neat looking instrument, and are easily kept clean. Their forms may be multiplied indefinitely, and there is no conformation of the jaw or teeth, or both, for which a suitable cup may not be made with this material in a few hours. For the want of a suitable cup in unusual cases, the operator is too often forced to content himself with an imperfect impression, and trust to trimming and guessing

